# Supplementary material for: Clinical and Prognostic Value of Antigen-Presenting Cells with PD-L1/PD-L2 Expression in Ovarian Cancer Patients
Source: Int J Mol Sci. 2021 Oct 26;22(21):11563. doi: 10.3390/ijms222111563 (PMC8583913; doi:10.3390/ijms222111563)
Supplement: Supplementary file 1 [file ijms-22-11563-s001.zip › ijms-1424203-supplementary.pdf]

Clinical and Prognostic Value of Antigen-Presenting Cells with PD-L1/PD-L2 Expression in Ovarian Cancer Patients

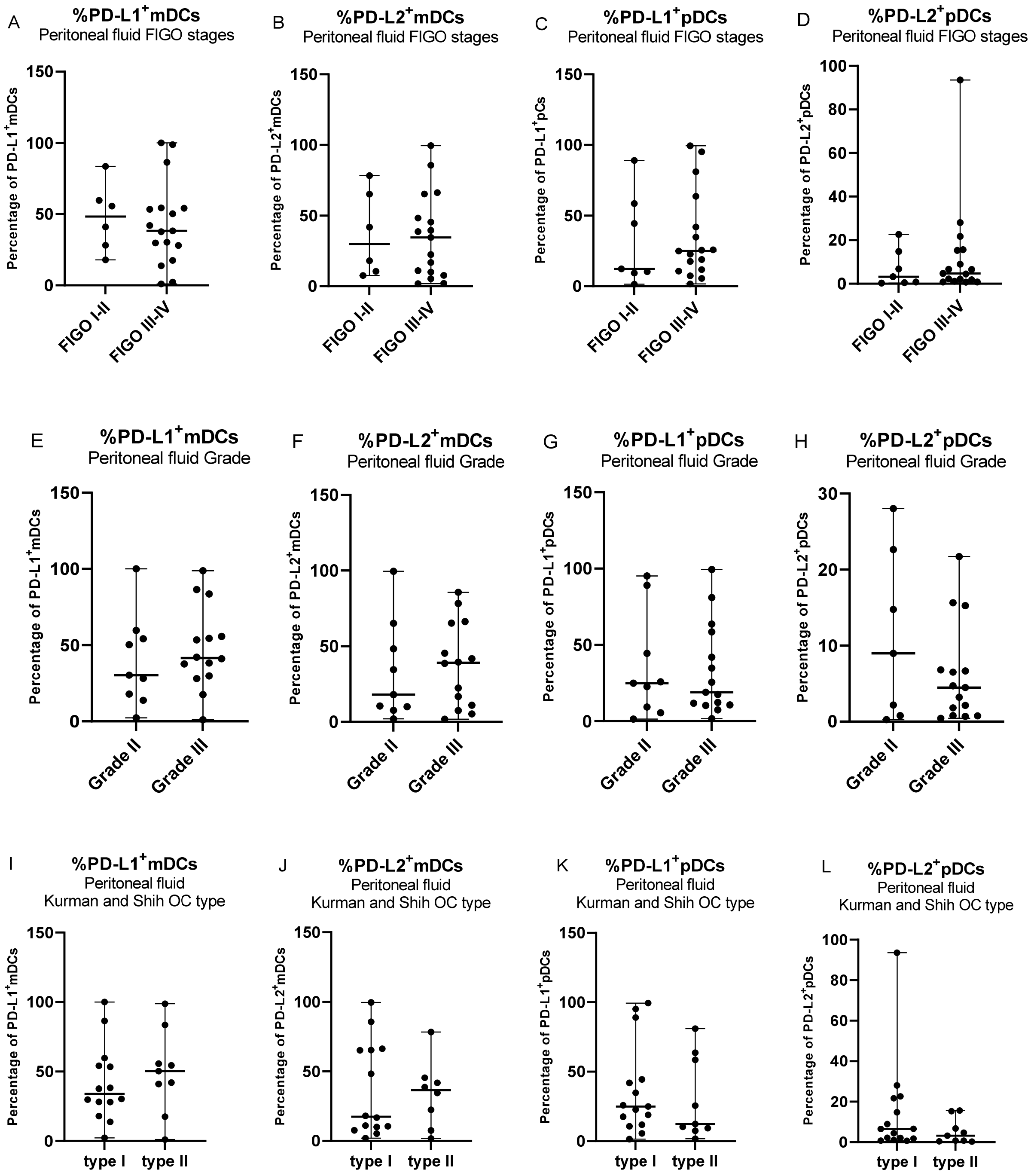

Figure S1. Distribution of mDCs and pDCs with PD-L1 and PD-L2 expression in peritoneal fluid of ovarian cancer patients with different FIGO stages **(A–D)**, grade **(E–H)**, and OC types **(I–L)**.

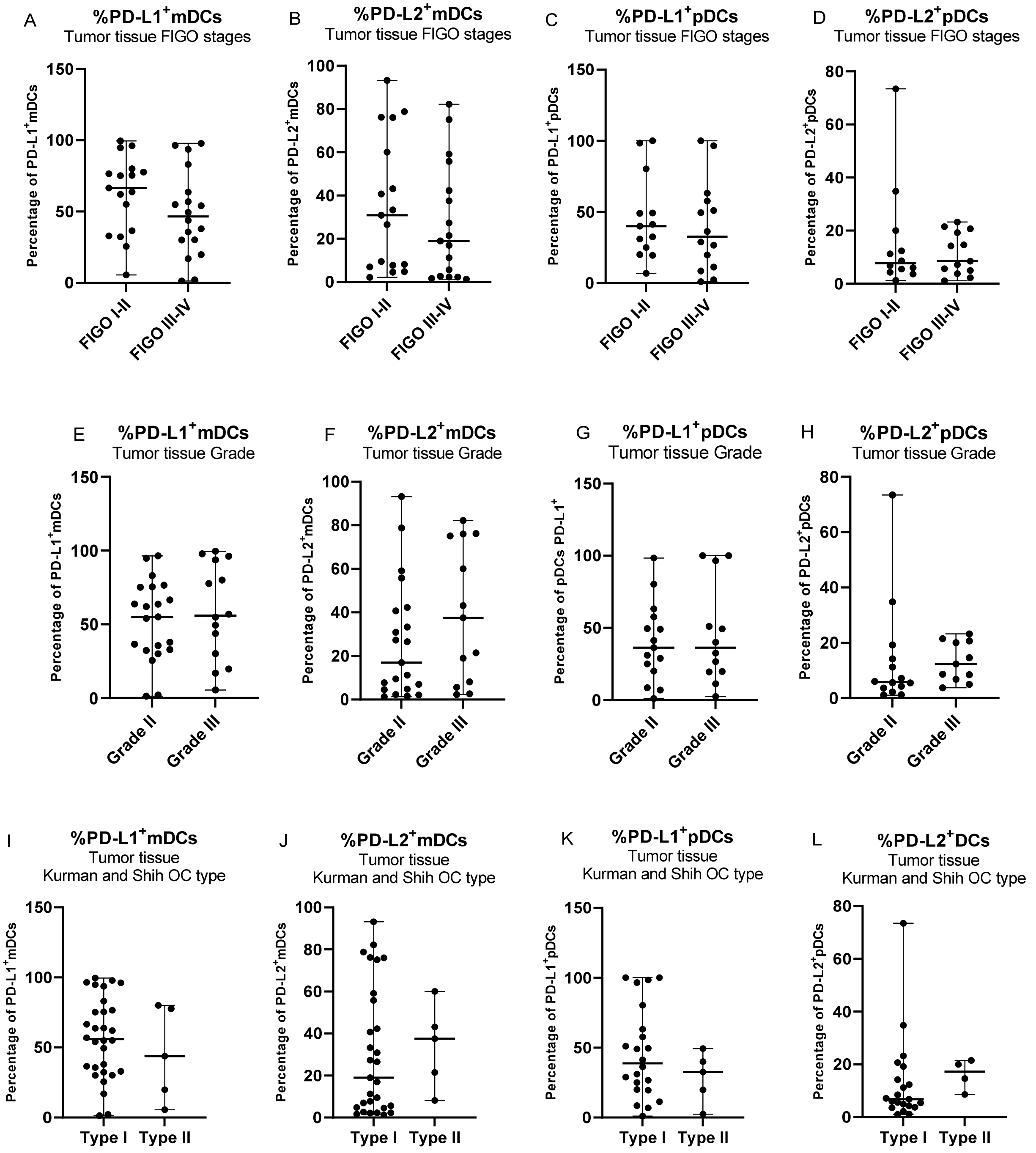

Figure S2. Distribution of mDCs and pDCs with PD-L1 and PD-L2 expression in tumor tissue of ovarian cancer patients with different FIGO stages **(A–D)**, grade **(E–H)**, and OC types **(I–L)**.

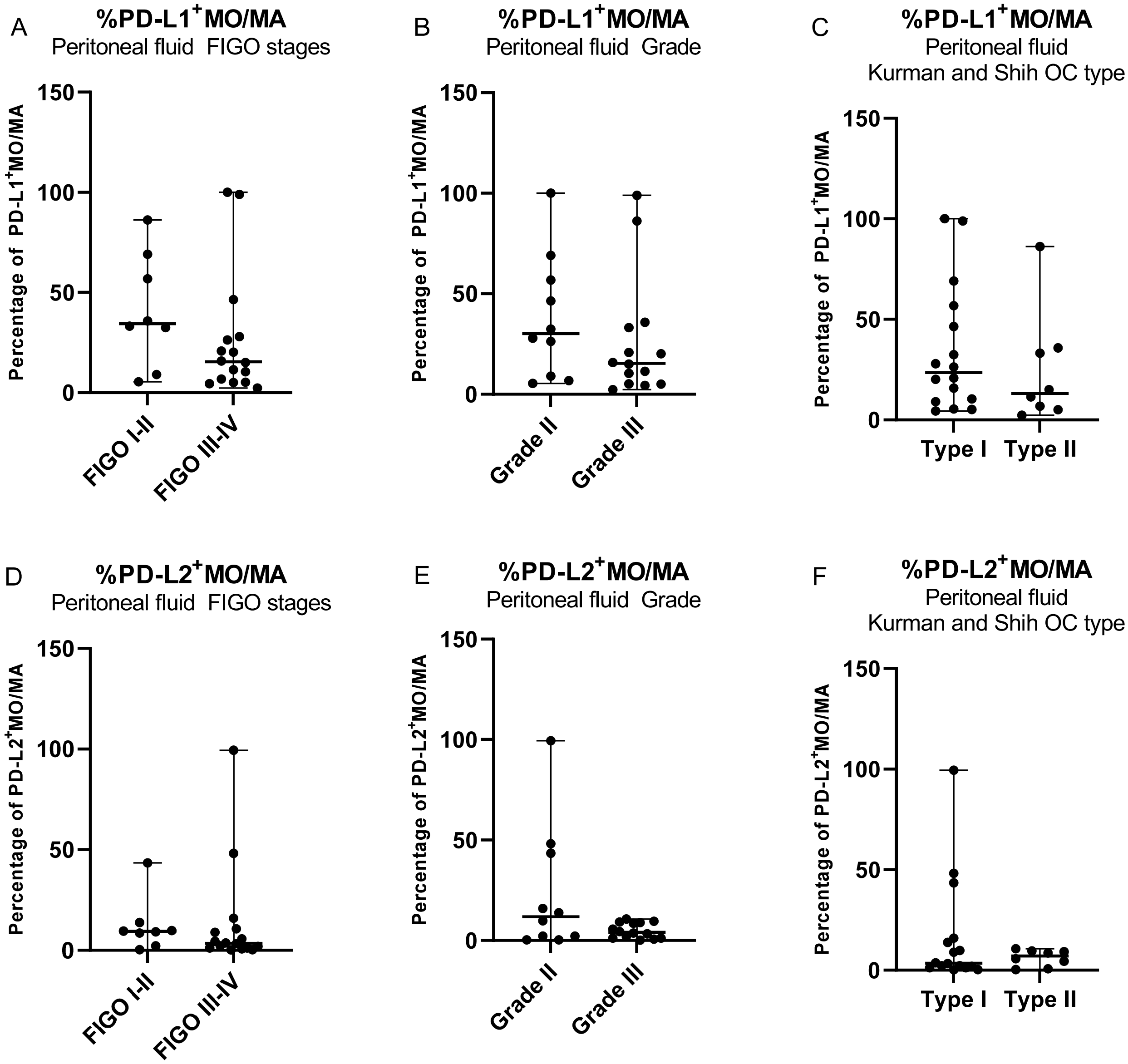

Figure S3. Distribution of MO/MA with PD-L1 and PD-L2 expression in peritoneal fluid of ovarian cancer patients with different FIGO stages **(A, D)**, grade **(B, E)**, and OC types **(C, F)**.

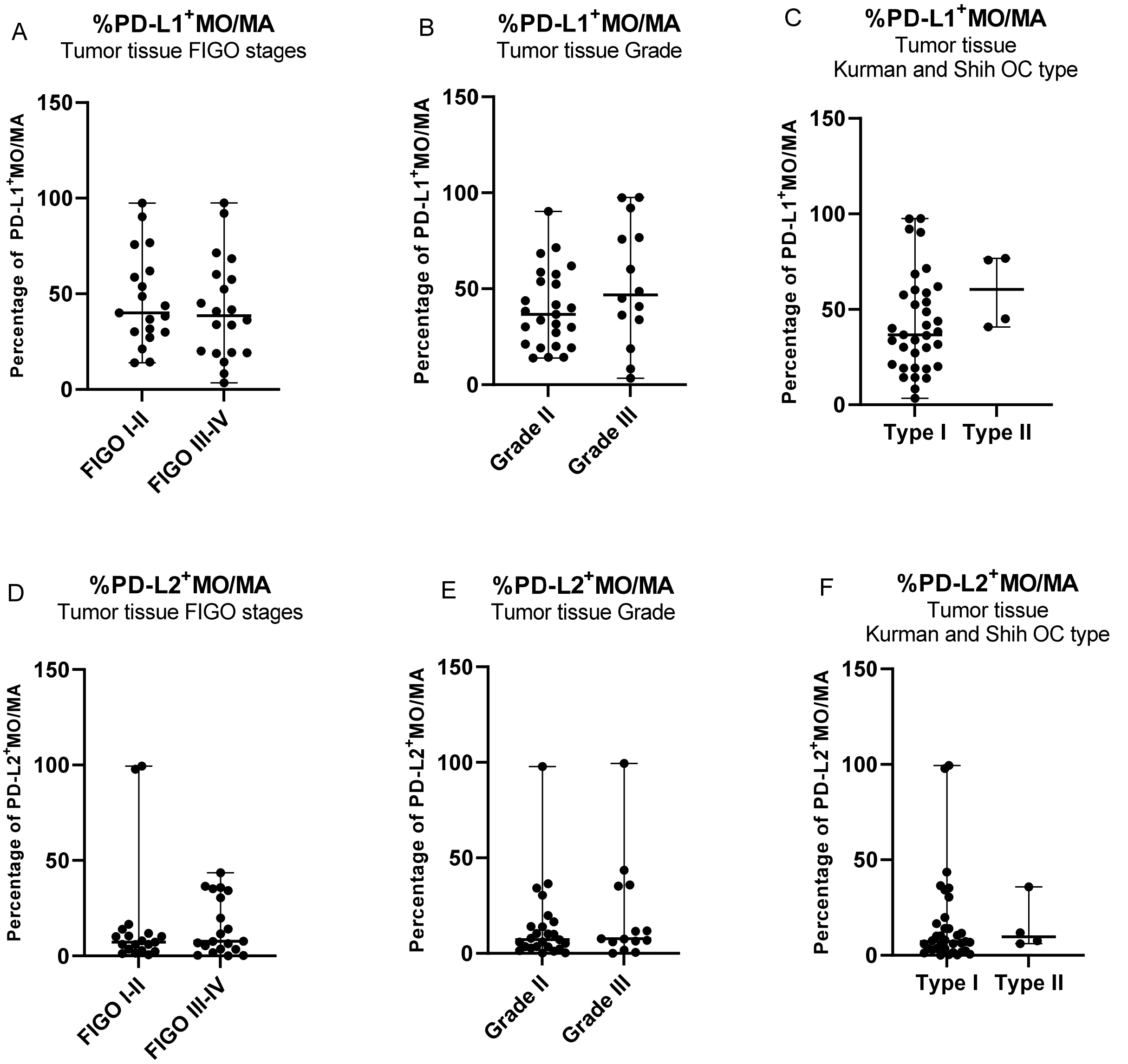

Figure S4. Distribution of MO/MA with PD-L1 and PD-L2 expression in tumor tissue of ovarian cancer patients with different FIGO stages **(A, D)**, grade **(B E)**, and OC types **(C, F)**.
